# Supplementary material for: Distinct roles of monkey OFC-subcortical pathways in adaptive behavior
Source: bioRxiv. 2023 Nov 17:2023.11.17.567492. Preprint. [Version 1] doi: 10.1101/2023.11.17.567492 (PMC10705585; doi:10.1101/2023.11.17.567492)
Supplement: Supplement 1 [file NIHPP2023.11.17.567492V1-supplement-1.pdf]

bioRxiv preprint doi: https://doi.org/10.1101/2023.11.17.567492; this version posted November 17, 2023. The copyright holder for this preprint (which was not certified by peer review) is the author/funder, who has granted bioRxiv a license to display the preprint in perpetuity. It is made available under aCC-BY 4.0 International license.

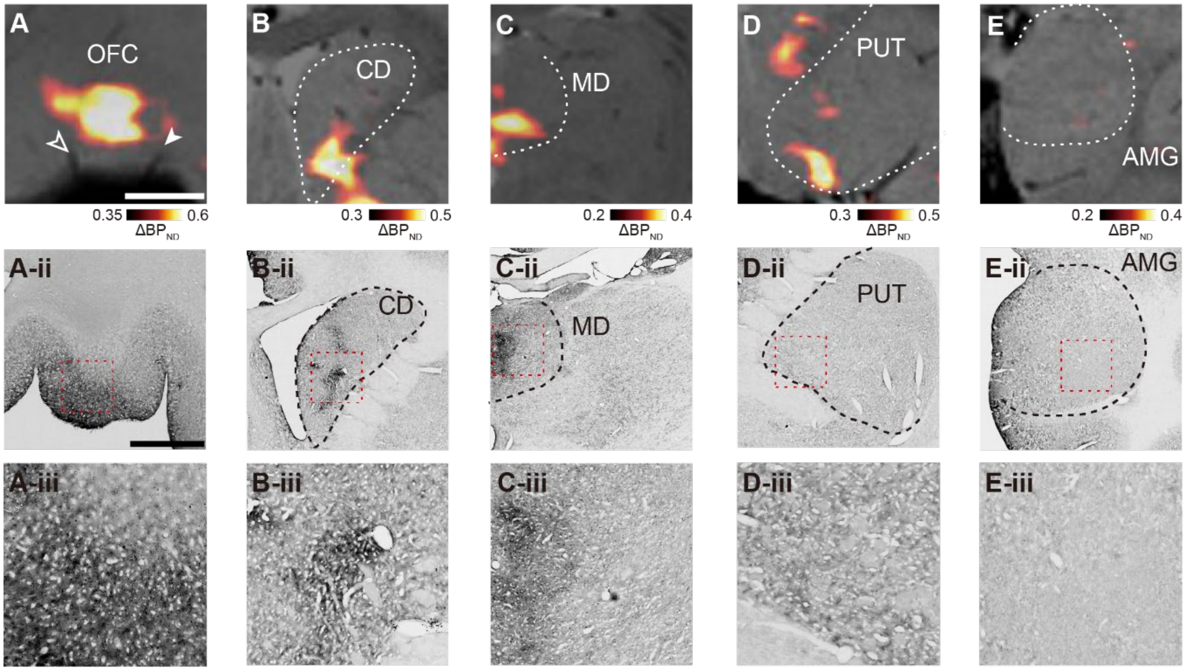

Fig. S1 | Expression of hM4Di in the OFC and its terminal sites.

(**A** to **E**) *In vivo* visualization of hM4Di expression in the OFC (**A**), rmCD (**B**), MDm (**C**), putamen (**D**), and amygdala (**E**) obtained from Mk#2. Images are coronal PET contrasts showing specific binding of [^{11}C]DCZ (contrast: after the introduction of hM4Di minus before the introduction), overlayed by MR images from Mk#2. The middle row visualizes corresponding DAB-stained sections showing immunoreactivity against a reporter protein (AcGFP), and the bottom row shows an enlarged view of the areas marked with red rectangles. Scale bars: 5 mm.

bioRxiv preprint doi: https://doi.org/10.1101/2023.11.17.567492; this version posted November 17, 2023. The copyright holder for this preprint (which was not certified by peer review) is the author/funder, who has granted bioRxiv a license to display the preprint in perpetuity. It is made available under aCC-BY 4.0 International license.

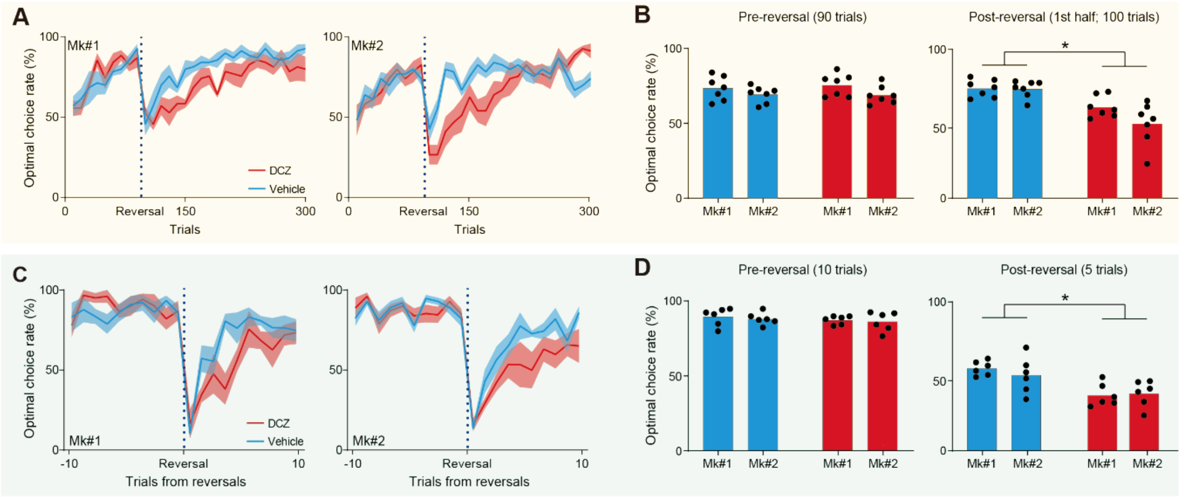

Fig. S2 | Effects of chemogenetic silencing of the bilateral OFC in each monkey.

(**A**) Behavioral performance for Mk#1 (left) and Mk#2 (right) on the NOVEL task (N = 7 for each treatment). (**B**) Optimal choice rate for the pre-reversal phase (left, 90 trials; two-way ANOVA, treatment, $F_{(1,24)} = 0.11$, $p = 0.74$; subject, $F_{(1,24)} = 4.4$, $p = 4.6 \times 10^{-2}$; interaction, $F_{(1,24)} = 0.21$, $p = 0.65$) and the 1st half of the post-reversal phase (right, 100 trials, treatment, $F_{(1,24)} = 28.0$, $p = 2.0 \times 10^{-5}$; subject, $F_{(1,24)} = 3.0$, $p = 0.10$; interaction, $F_{(1,24)} = 2.5$, $p = 0.12$). (**C**) Behavioral performance for Mk#1 (left) and Mk#2 (right) on the FAMILIAR task (N = 6 for each treatment). (**D**) Optimal choice rate for the pre-reversal phase (left, 10 trials; two-way ANOVA, treatment, $F_{(1,20)} = 0.36$, p = 0.56; subject, $F_{(1,20)} = 0.9$, $p = 0.34$; interaction, $F_{(1,20)} = 0.009$, p = 0.93) and the post-reversal phase (right, 5 trials, treatment, $F_{(1,20)} = 17.8$, $p = 4.2 \times 10^{-4}$; subject, $F_{(1,20)} = 0.25$, $p = 0.62$; interaction, $F_{(1,20)} = 0.68$, $p = 0.42$). Asterisks: $p < 0.05$ for significant main effect of treatment.

bioRxiv preprint doi: https://doi.org/10.1101/2023.11.17.567492; this version posted November 17, 2023. The copyright holder for this preprint (which was not certified by peer review) is the author/funder, who has granted bioRxiv a license to display the preprint in perpetuity. It is made available under aCC-BY 4.0 International license.

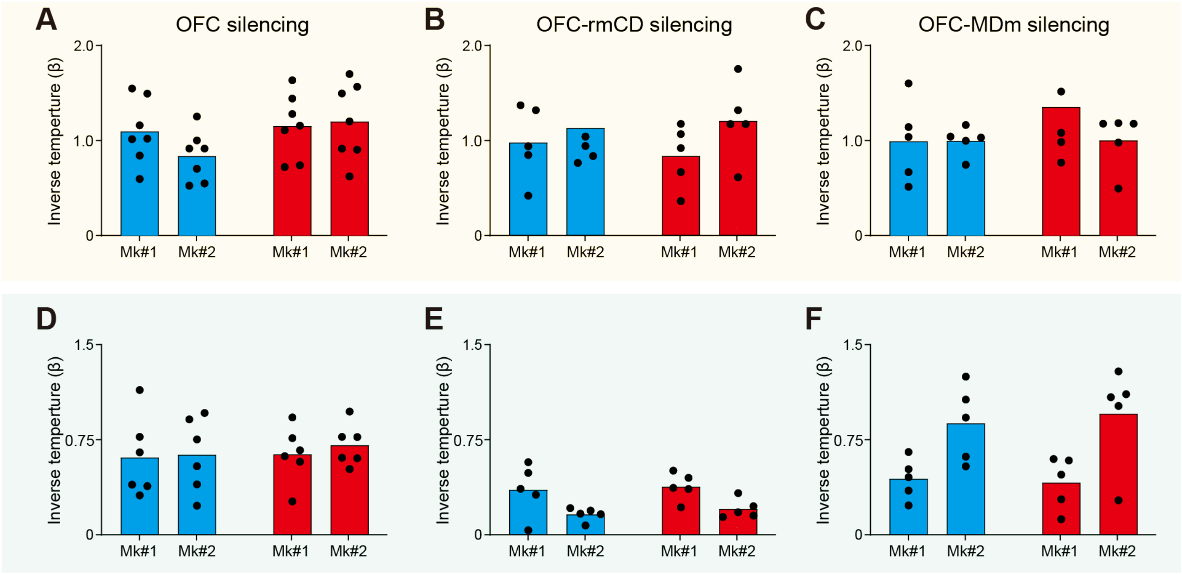

Fig. S3 | Estimated inverse temperature by model fitting analysis.

(**A** to **C**) Inverse temperatures estimated by fitting performance on the NOVEL task to the EXP model after silencing the OFC (**A**) (two-way ANOVA, treatment, $F_{(1,24)} = 2.7$, $p = 0.11$; subject, $F_{(1,24)} = 0.69$, $p = 0.41$; interaction, $F_{(1,24)} = 1.4$, $p = 0.24$), the OFC-rmCD pathway (**B**) (treatment, $F_{(1,16)} = 0.03$, $p = 0.86$; subject, $F_{(1,16)} = 1.9$, $p = 0.18$; interaction, $F_{(1,16)} = 0.33$, $p = 0.58$), or the OFC-MDm pathway (**C**) (treatment, $F_{(1,16)} = 0.94$, $p = 0.35$; subject, $F_{(1,16)} = 0.85$, $p = 0.37$; interaction, $F_{(1,16)} = 0.87$, $p = 0.37$) for control vehicle (cyan) and DCZ treatment (red) in each monkey. Only data for the post-reversal phase are shown. (**D** to **F**) Inverse temperatures estimated by fitting to the performance on the FAMILIAR task to the INF model after silencing of the OFC (**D**) (two-way ANOVA, treatment, $\mathrm{F}_{(1,20)} = 0.21$, $\mathrm{p} = 0.65$; subject, $F_{(1,20)} = 0.24$, $\mathrm{p} = 0.63$; interaction, $F_{(1,20)} = 0.054$, $\mathrm{p} = 0.82$), the OFC-rmCD pathway (**E**) (treatment, $F_{(1,16)} = 0.39$, $p = 0.54$; subject, $F_{(1,16)} = 11.0$, $p = 4.5 \times 10^{-3}$; interaction, $F_{(1,16)} = 0.026$, $p = 0.87$), or the OFC-MDm pathway (**F**) (treatment, $F_{(1,16)} = 0.034$, $p = 0.86$; subject, $F_{(1,16)} = 15.4$, $p = 1.2 \times 10^{-3}$; interaction, $F_{(1,16)} = 0.17$, $p = 0.68$).

bioRxiv preprint doi: https://doi.org/10.1101/2023.11.17.567492; this version posted November 17, 2023. The copyright holder for this preprint (which was not certified by peer review) is the author/funder, who has granted bioRxiv a license to display the preprint in perpetuity. It is made available under aCC-BY 4.0 International license.

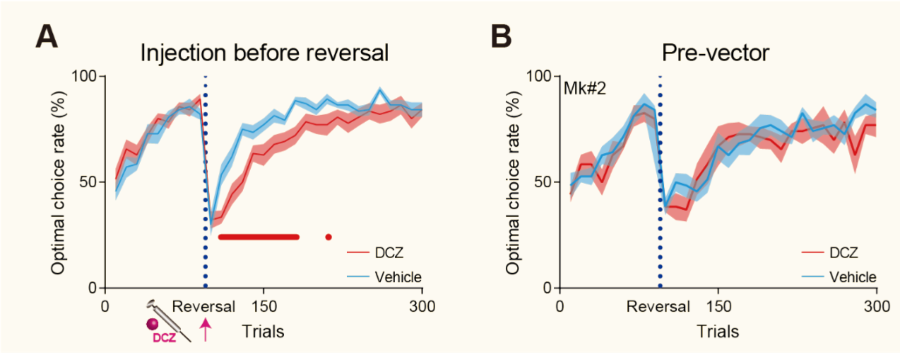

Fig. S4 | Effects of intramuscular DCZ administration just before the reversal and without hM4Di expression on the NOVEL task performance.

(**A**) Behavioral performance on the NOVEL task (N = 7 for each treatment in each monkey) when DCZ was administered intramuscularly just before reversal. The task was interrupted for 5 min and DCZ was administered at the start of the 5-min break. (**B**) Behavioral performance on the NOVEL task (N = 7 for each treatment, Mk#2) before the introduction of hM4Di.

bioRxiv preprint doi: https://doi.org/10.1101/2023.11.17.567492; this version posted November 17, 2023. The copyright holder for this preprint (which was not certified by peer review) is the author/funder, who has granted bioRxiv a license to display the preprint in perpetuity. It is made available under aCC-BY 4.0 International license.

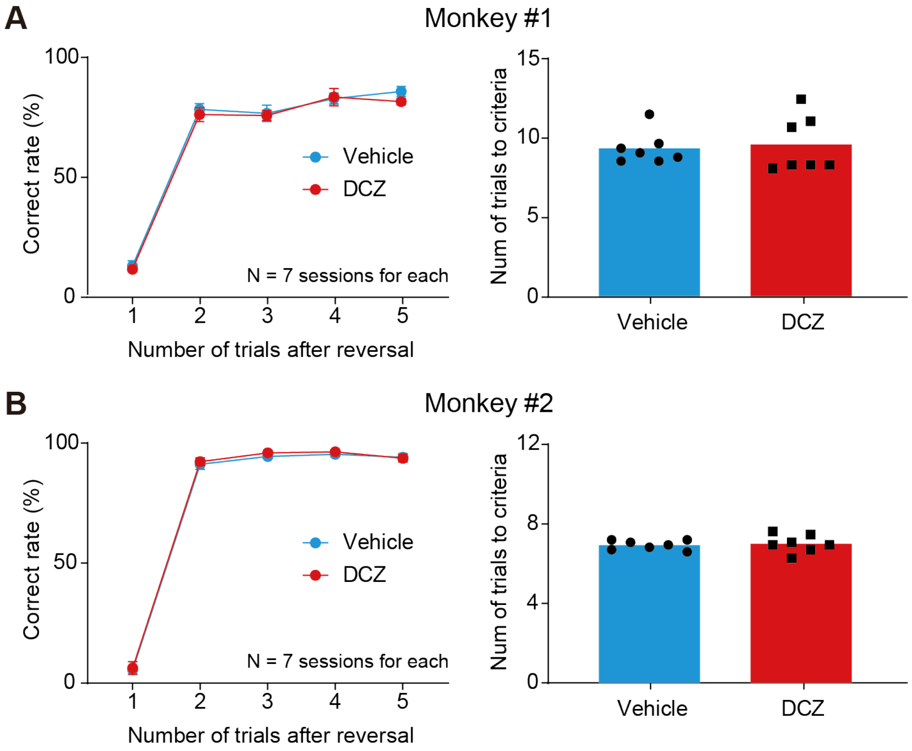

Fig. S5 | Effect of chemogenetically silencing the bilateral OFC on the two-arm reversal learning task.

(**A** and **B**) Correct rate as a function of the number of trials after reversal (left) and the number of trials to reach criteria (right) for Mk#1 (**A**) and Mk#2 (**B**). There was no significant difference between vehicle and DCZ injection (Two-tailed Welch's t-test, Mk#1, $t_{9.7} = 0.33$, $p = 0.75$; Mk#2, $t_{9.1} = 0.37$, $p = 0.72$).

bioRxiv preprint doi: https://doi.org/10.1101/2023.11.17.567492; this version posted November 17, 2023. The copyright holder for this preprint (which was not certified by peer review) is the author/funder, who has granted bioRxiv a license to display the preprint in perpetuity. It is made available under aCC-BY 4.0 International license.

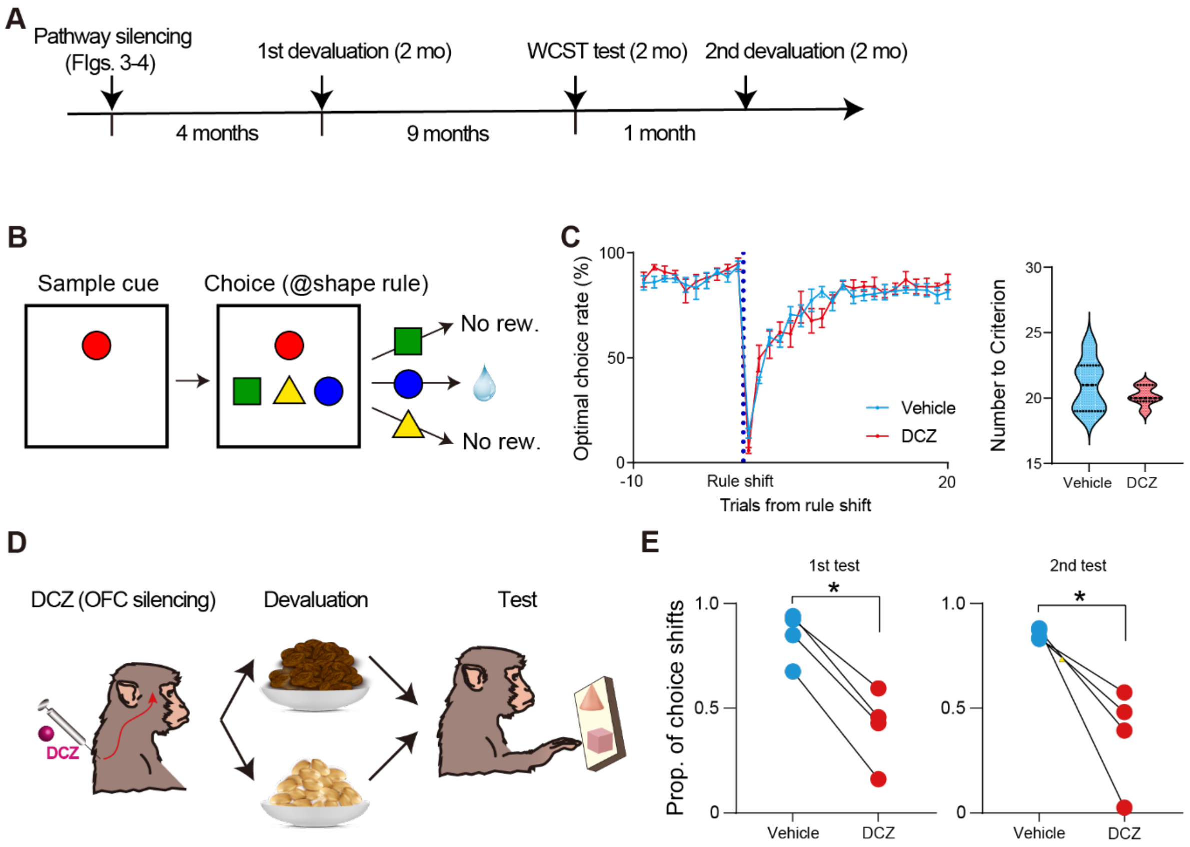

Fig. S6 | Effect of chemogenetically silencing the bilateral OFC on performance for the Wisconsin Card Sorting Test and the devaluation test.

Schema of the Wisconsin Card Sorting Test. (**C**) Optimal choice rate (N = 6 for each treatment) as a function of trials for rule shift (left) and the number of trials to reach the criterion for rule shift (right). There was no significant difference in the number needed to reach criterion between vehicle and DCZ injection conditions (Two-tailed Welch's t-test, $t_{6.4} = 0.96$, $p = 0.37$). The horizontal lines in each violin plot show the quartiles of the distributions. (**D**) Schema for the devaluation test. (**E**) Performance on the devaluation test for the 1st (left) and 2nd schedule (right), respectively (N = 4 for each treatment for both schedules). There was a significant difference in performance between vehicle and DCZ injections for both schedules (Two-tailed Welch's t-test, 1st, $t_{3.1} = 4.0$, $p = 2.7 \times 10^{-2}$).

bioRxiv preprint doi: https://doi.org/10.1101/2023.11.17.567492; this version posted November 17, 2023. The copyright holder for this preprint (which was not certified by peer review) is the author/funder, who has granted bioRxiv a license to display the preprint in perpetuity. It is made available under aCC-BY 4.0 International license.

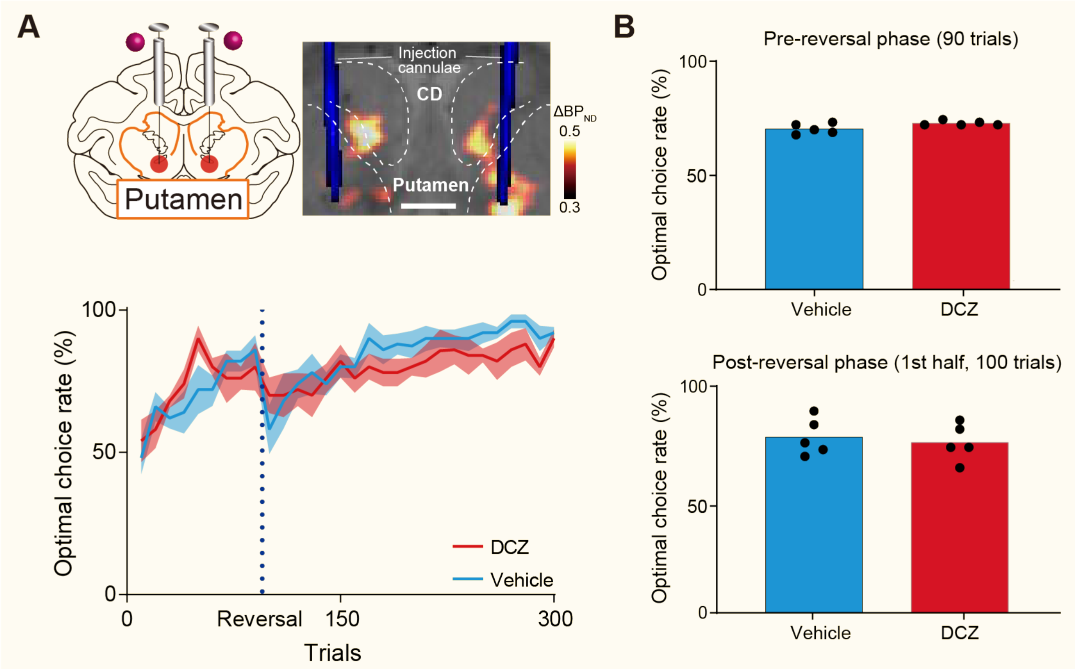

Fig. S7 | Effects of chemogenetically silencing the OFC-mPut pathway on NOVEL task performance.

(**A**) Chemogenetically silencing the OFC-mPut pathway by local DCZ infusion into bilateral mPut, specifically at hM4Di-positive OFC terminals sites (top), and behavioral performance (bottom). N = 5 sessions for each treatment. Conventions are the same as in Fig. 2. (**B**) Averaged optimal choice rate in different task phases (Two-tailed Welch's t-test, pre-reversal phase: $t_{5.4} = 2.2$, $p = 0.08$; 1st half of post-reversal phase: $t_{8.0} = 0.46$, $p = 0.66$).

bioRxiv preprint doi: https://doi.org/10.1101/2023.11.17.567492; this version posted November 17, 2023. The copyright holder for this preprint (which was not certified by peer review) is the author/funder, who has granted bioRxiv a license to display the preprint in perpetuity. It is made available under aCC-BY 4.0 International license.

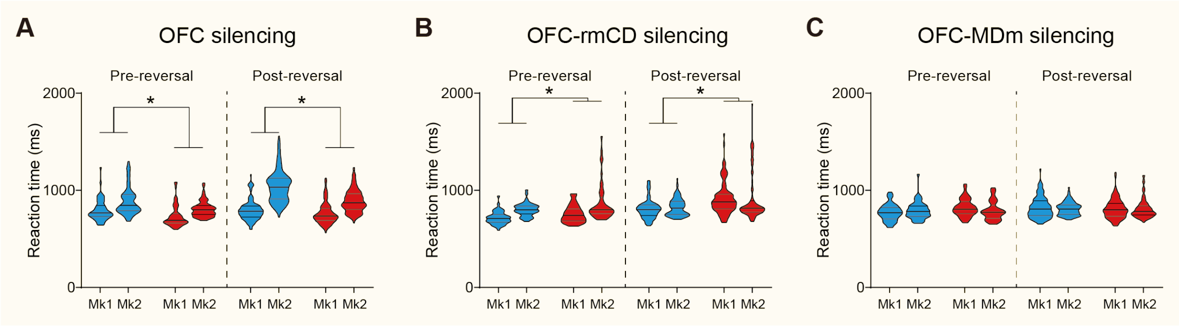

Fig. S8 | Effects of chemogenetically silencing the OFC, OFC-rmCD pathway, and OFC-MDm pathway on reaction time during the NOVEL task.

(**A** to **C**) Reaction time in the pre-reversal phase (left) and post-reversal phase (right) after silencing the OFC (**A**), OFC-rmCD pathway (**B**), and OFC-MDm pathway (**C**) with DCZ treatment (red) or after control vehicle (cyan, no silencing) in each monkey. In both task phases, reaction times increased significantly after OFC silencing (pre-reversal phase: treatment, $F_{(1,248)} = 25.3$, $p = 9.5 \times 10^{-7}$; subject, $F_{(1,248)} = 38.0$, $p = 2.8 \times 10^{-9}$; interaction, $F_{(1,248)} = 0.62$, $p = 0.43$; post-reversal phase: treatment, $F_{(1,584)} = 74.1$, $p = 6.8 \times 10^{-17}$; subject, $F_{(1,584)} = 325.7$, $p = 3.4 \times 10^{-58}$; interaction, $F_{(1,584)} = 74.1$, $p = 7.3 \times 10^{-10}$), decrease significantly after silencing the OFC-rmCD pathway (pre-reversal phase: treatment, $F_{(1,176)} = 10.5$, $p = 1.5 \times 10^{-3}$; subject, $F_{(1,176)} = 32.6$, $p = 4.7 \times 10^{-8}$; interaction, $F_{(1,176)} = 0.77$, $p = 0.38$; post-reversal phase: treatment, $F_{(1,416)} = 37.8$, $p = 1.9 \times 10^{-9}$; subject, $F_{(1,416)} = 0.34$, $p = 0.56$; interaction, $F_{(1,416)} = 0.39$, $p = 0.53$), and did not change after silencing the OFC-MDm pathway (pre-reversal phase: treatment, $F_{(1,176)} = 2.7$, $p = 0.10$; subject, $F_{(1,176)} = 0.14$, $p = 0.71$; interaction, $F_{(1,176)} = 3.7$, $p = 0.06$; post-reversal phase: treatment, $F_{(1,416)} = 0.76$, $p = 0.38$; subject, $F_{(1,416)} = 0.22$, $p = 0.64$; interaction, $F_{(1,416)} = 0.58$, p = 0.45). The horizontal lines in each violin plot show the quartiles of the distributions. Asterisks: $p < 0.05$ for significant main effect of treatment.

bioRxiv preprint doi: https://doi.org/10.1101/2023.11.17.567492; this version posted November 17, 2023. The copyright holder for this preprint (which was not certified by peer review) is the author/funder, who has granted bioRxiv a license to display the preprint in perpetuity. It is made available under aCC-BY 4.0 International license.

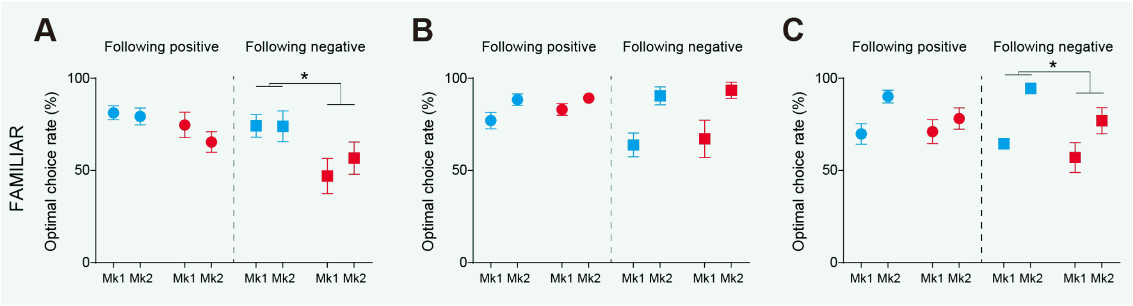

Fig. S9 | The effects of silencing the OFC, OFC-rmCD pathway, and OFC-MDm pathway on the sensitivity to past outcomes during the FAMILIAR task.

(**A** to **C**) Averaged optimal choice rates for trials after the reversal in the FAMILIAR task following positive (left) and negative (right) outcomes after OFC silencing (**A**) (positive: treatment, $F_{(1,20)} = 3.7$, p = 0.069; subject, $F_{(1,20)} = 1.1$, $p = 0.31$; interaction, $F_{(1,20)} = 0.47$, $p = 0.50$; negative: treatment, $F_{(1,20)} = 7.2$, $p = 1.4 \times 10^{-2}$; subject, $F_{(1,20)} = 0.33$, $p = 0.57$; interaction, $F_{(1,20)} = 0.36$, $p = 0.55$), OFC-rmCD silencing (**B**) (positive: treatment, $F_{(1,16)} = 1.0$, $p = 0.32$; subject, $F_{(1,16)} = 6.6$, $p = 2.1 \times 10^{-2}$; interaction, $F_{(1,16)} = 0.58$, $p = 0.46$; negative: treatment, $F_{(1,16)} = 0.21$, $p = 0.65$; subject, $F_{(1,16)} = 15.1$, 1.3×10^{-3}; interaction, $F_{(1,16)} = 0.0004$, $p = 0.99$), and OFC-MDm silencing (**C**) (positive: treatment, $F_{(1,16)} = 0.40$, $p = 0.54$; subject, $F_{(1,16)} = 2.6$, $p = 0.13$; interaction, $F_{(1,16)} = 0.10$, $p = 0.76$; negative: treatment, $F_{(1,16)} = 6.0$, $p = 2.6 \times 10^{-2}$; subject, $F_{(1,16)} = 18.0$, $p = 6.0 \times 10^{-4}$; interaction, $F_{(1,16)} = 0.17$, $p = 0.68$).
